# A Study on Hollow Viscus Perforation in a Tertiary Care Hospital in South India

**DOI:** 10.7759/cureus.71500

**Published:** 2024-10-14

**Authors:** Magesh Chandran, Aiswerya Shankar, Kuberan Krishnan, Madan Sundar, Mahesh K G

**Affiliations:** 1 Surgery, Bharath Institute of Higher Education and Research, Chennai, IND; 2 General Surgery, Sree Balaji Medical College & Hospital, Chennai, IND; 3 General Surgery, Bharath Institute of Higher Education and Research, Chennai, IND

**Keywords:** air under the diaphragm, hollow viscus perforation, perforation, peritonitis, sepsis

## Abstract

Introduction

Hollow viscus perforation refers to the perforation of the gastrointestinal tract, including the stomach, intestines, or other hollow organs, leading to leakage of the contents into the peritoneal cavity. This paper aims to explore the disease burden of hollow viscus perforation in Sree Balaji Medical College & Hospital in Chennai, India, and its relation to patients’ age and sex and analyze the etiology of hollow viscus perforation.

Materials and methods

This single-center retrospective study was conducted between May 2022 and August 2023, with a sample size of 100 patients. Data were extracted from the hospital’s medical records based on the study’s parameters.

Results

Our study shows that among the study group, hollow viscus perforation was more common in males (n = 78; 78%) than females. The highest incidence was observed in the 41-50 age group (n = 35; 35%), with the mean age being 43 years. The most common cause of perforation was a duodenal ulcer. Wound infection was the most frequent postoperative complication, affecting less than one-third of the patients (n = 29; 29%), followed by pneumonia and acute respiratory distress syndrome. Although large intestinal pathology affected only eight patients in the sample, it showed a high case fatality rate, with one-quarter of the patients (n = 2; 25%) succumbing to the condition.

Conclusion

Hollow viscus perforation is a life-threatening condition that requires prompt recognition and treatment. The etiology is diverse, ranging from peptic ulcer disease and malignancy to trauma and inflammatory conditions. Early diagnosis and aggressive management are essential for improving the outcome for patients with this condition.

## Introduction

Acute abdomen or abdominal pain accounts for nearly four of every 10 hospital admissions, with most cases resulting from a perforated organ or one in the stage of imminent perforation [1-3]. The gastrointestinal (GI) tract begins with the stomach and continues as the duodenum, jejunum, and ileum, forming the small intestine, then leads through the appendix to the large intestine and finally the rectum. A breach in the lumen anywhere along this tract can lead to contamination of the organs, peritoneum, and abdominal cavity, causing infections, abscesses, and peritonitis [4]. The site of perforation and the degree of contamination play a crucial role in determining the severity of peritonitis [5,6].

The major causes of GI perforation can be divided into several subcategories. Perforation due to foreign bodies, either through ingestion of sharp objects or corrosive substances or penetrating trauma, can occur as the object passes through the GI tract. A volvulus hernia causes perforation due to ischemia and necrosis of the bowel, whereas benign or malignant tumors such as GI stromal tumors, and lymphoma can cause perforation due to extrinsic bowel obstruction. An intrinsic bowel obstruction could result from strictures seen in Crohn’s disease, diverticulitis, and appendicitis, where increased intraluminal pressure may lead to rupture of the bowel, particularly through a closed-loop mechanism [7-9].

Some of the most substantial causes of GI perforation are peptic ulcer disease (PUD) and Crohn’s disease, both of which can compromise the structural integrity of the GI tract. Other causes include ischemia from shock, hypotension, or thromboembolism. In addition, conditions such as typhoid fever and tuberculosis can cause perforation of the ileum due to ulceration from the infection. Several known risk factors increase the likelihood of developing hollow viscus perforation, including chronic non-steroidal anti-inflammatory drug (NSAID) use, chronic alcoholism or smoking, old age, and an immunocompromised state. Of all investigations, the abdominal CT is the most sensitive and specific for identifying the exact cause of hollow viscus perforation [10]. Most cases that show signs of peritonitis and sepsis require definitive surgery, but those without signs of peritonitis can be managed conservatively [11,12].

The aim and the primary objective of this study were to understand the disease burden of hollow viscus perforation in a tertiary care center and its relation to patients’ age and sex. The secondary objective was to analyze the etiology of hollow viscus perforation.

## Materials and methods

Study design

This is a single-center retrospective study conducted between May 2022 and August 2023 (one year and three months) in the Department of General Surgery, the Department of Surgical Gastroenterology, and the Emergency Department of Sree Balaji Medical College & Hospital, Chennai, India. Data were obtained from hospital medical records within the specified time frame. The sample size consisted of 100 patients (using convenience sampling) diagnosed with hollow viscus perforation.

Ethical considerations

Informed consent (verbal) was obtained from all participants included in the study. The study was designed to ensure that the rights of the participants were protected and that the research adhered to ethical standards. The study was conducted in compliance with ethical guidelines for human research after obtaining appropriate approval.

Study criteria

Participants were selected based on specific inclusion and exclusion criteria. The inclusion criteria ensured this study involved patients who presented with abdominal pain, distension, or vomiting, were between 18 and 65 years of age of either sex, and had an identified traumatic or atraumatic hollow viscus perforation. The exclusion criteria eliminated patients with perforations of the genitourinary tract and those with ruptured ectopic pregnancies from this study.

Methods

After extracting data from the hospital medical records, data was compiled. All patients had undergone a thorough clinical examination, preliminary laboratory investigations, and abdominal computed tomography (CT) scans. After active resuscitation and stabilization, all patients were started on antibiotic coverage and taken for an exploratory or lower midline laparotomy. Age, sex, and etiology for each case of perforation were extracted from the records and analyzed.

Statistical analysis

Statistical analysis was performed with mean and standard deviation to determine the demographic distribution of hollow viscus perforation among patients at our tertiary care hospital. This study examined age and sex distribution and the etiology of hollow viscus perforation.

## Results

Table 1 shows the age and sex distribution among our patients with hollow viscus perforation. Over one-third of the cases (n = 35; 35%) were between the ages of 41 and 50 years. The second highest incidence was 23 patients in the 31-40 age group. The mean age in this study was found to be 43 years. In our entire study, over three-quarters of the patients (n = 78; 78%) were males, with a male-to-female ratio of 3.5:1. This showed that the relative incidence of hollow viscus perforation was higher in males.

**Table 1:** Age and sex distribution of perforation in our study

Age (years)	Number of male patients (N = 78)	Number of female patients (N = 22)	Total percentage (N = 100)
18–20	1	3	4%
21–30	6	2	8%
31–40	18	5	23%
41–50	30	5	35%
51–60	14	6	20%
>60	7	3	10%
Total (ratio M:F = 3.5:1)	78	22	100%

**Figure 1:**
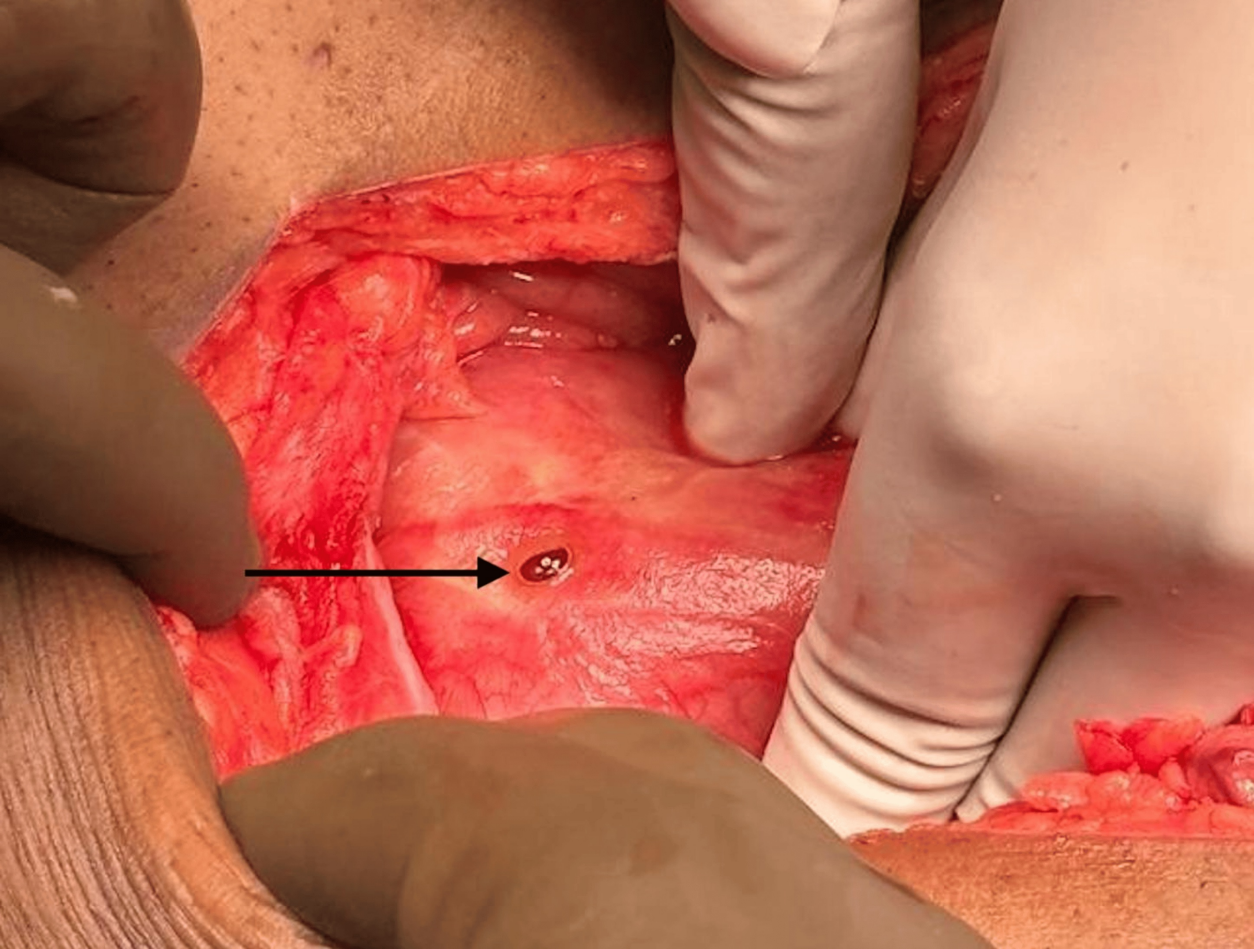
Representative image showing duodenal perforation(pointer)

Table 2 shows the organ-specific incidence based on the various causes of perforation. It was found that more than half of the patients (n = 51; 51%) suffered from gastric or duodenal ulcers, with duodenal ulcers being the leading cause of perforation (Figure 1). Of these 31 cases, 14 had a history of peptic ulcer disease (n = 14; 45%), three were biopsy-proven for a *Helicobacter pylori *infection, and six had a history of NSAID abuse (n = 6; 16%).

**Table 2:** Relative incidence of perforation in relation to the anatomical site and their causes

Site	Cause	Number of patients (N = 100)
Gallbladder	Acute cholecystitis	12
Stomach	Gastric ulcer	20
Duodenum	Duodenal ulcer	31
Appendix	Acute appendicitis with rupture	13
	Appendicolith leading to rupture	3
Small Intestine	Typhoid	4
	Stricture leading to perforation	5
	Perforation of Meckel’s diverticulum	2
	Traumatic blunt injury	2
Large Intestine	Perforation of sigmoid colon growth	5
	Caecal perforation due to ileocecal growth	3

**Figure 2:**
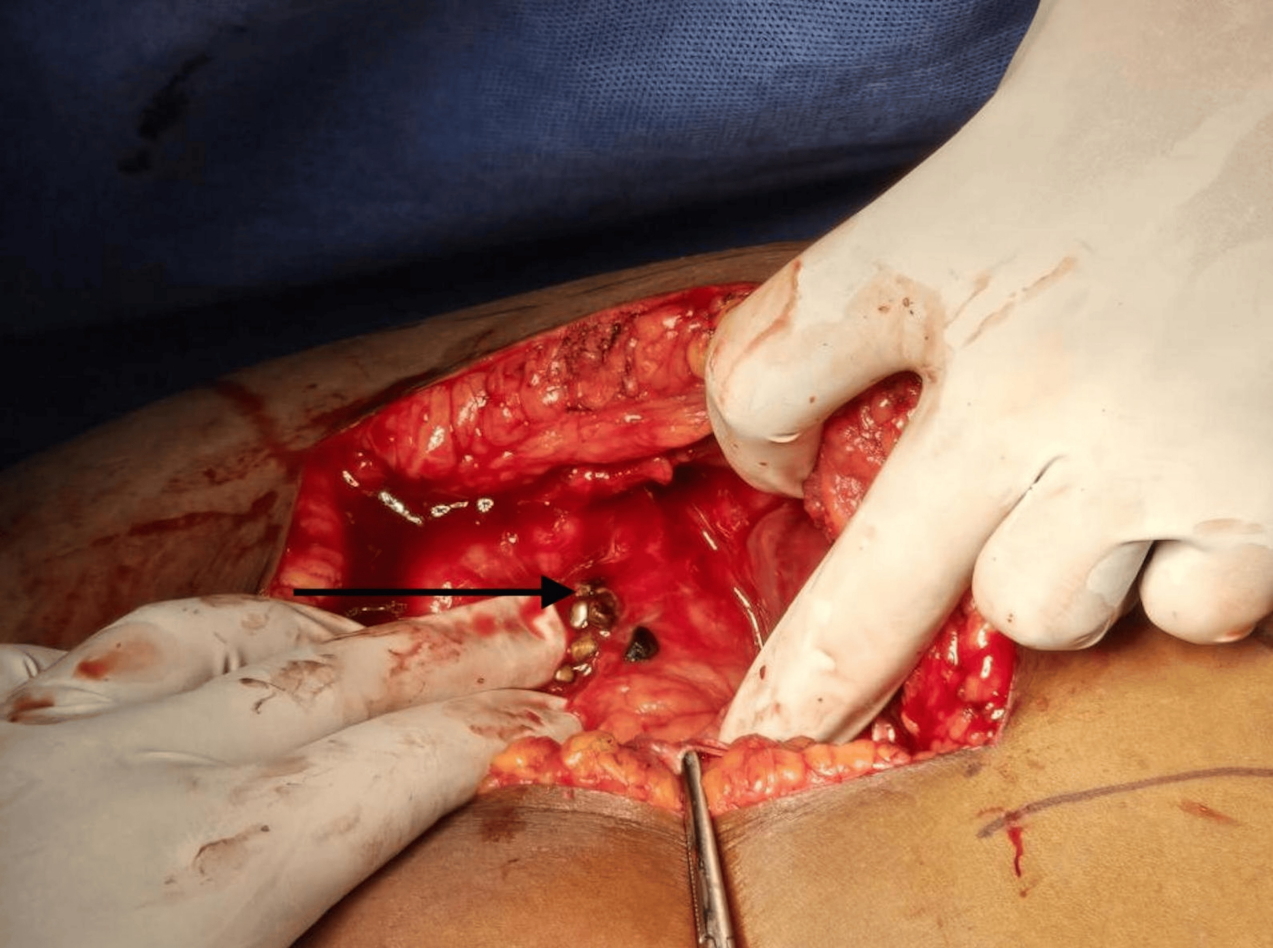
Representative image showing gallbladder perforation due to acute cholecystitis (pointer)

**Figure 3:**
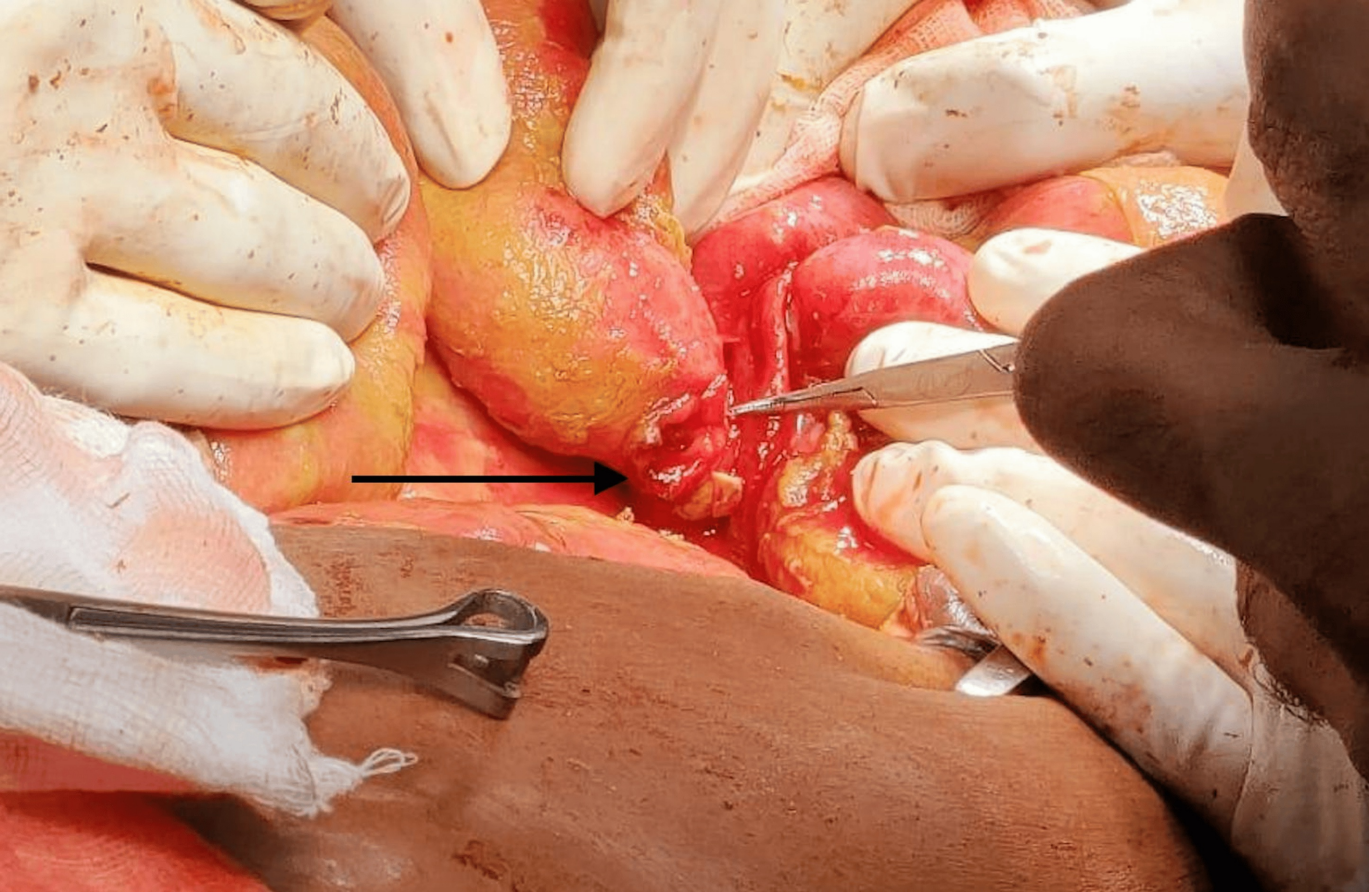
Representative image showing perforation in the small intestine near ileum (pointer)

Over one-tenth of the patients (n = 13; 13%) presented with a ruptured appendix. Twelve patients presented with acute cholecystitis (Figure 2) and 13 patients presented with small bowel pathology (Figure 3). Table 3 shows the various presentations associated with perforation. The majority of the patients with a gallbladder or stomach pathology most commonly presented with abdominal pain, vomiting, and tachycardia, whereas patients with duodenal pathology presented with abdominal pain, tachycardia, and hypotension concomitant with oliguria.

**Table 3:** Various presentations associated with perforation

Clinical features	Gallbladder (N = 12)	Stomach (N = 20)	Duodenum (N = 31)	Appendix (N = 16)	Small intestine (N = 13)	Large intestine (N = 8)
Abdominal pain	10	16	26	14	6	4
Nausea	6	10	9	10	-	-
Vomiting	6	13	5	9	2	2
Fever	4	-	10	8	-	-
Tachycardia	7	-	12	13	10	4
Hypotension	-	9	12	-	6	4
Localized peritonitis	4	-	7	7	-	-
Oliguria	-	-	12	6	2	5

**Figure 4:**
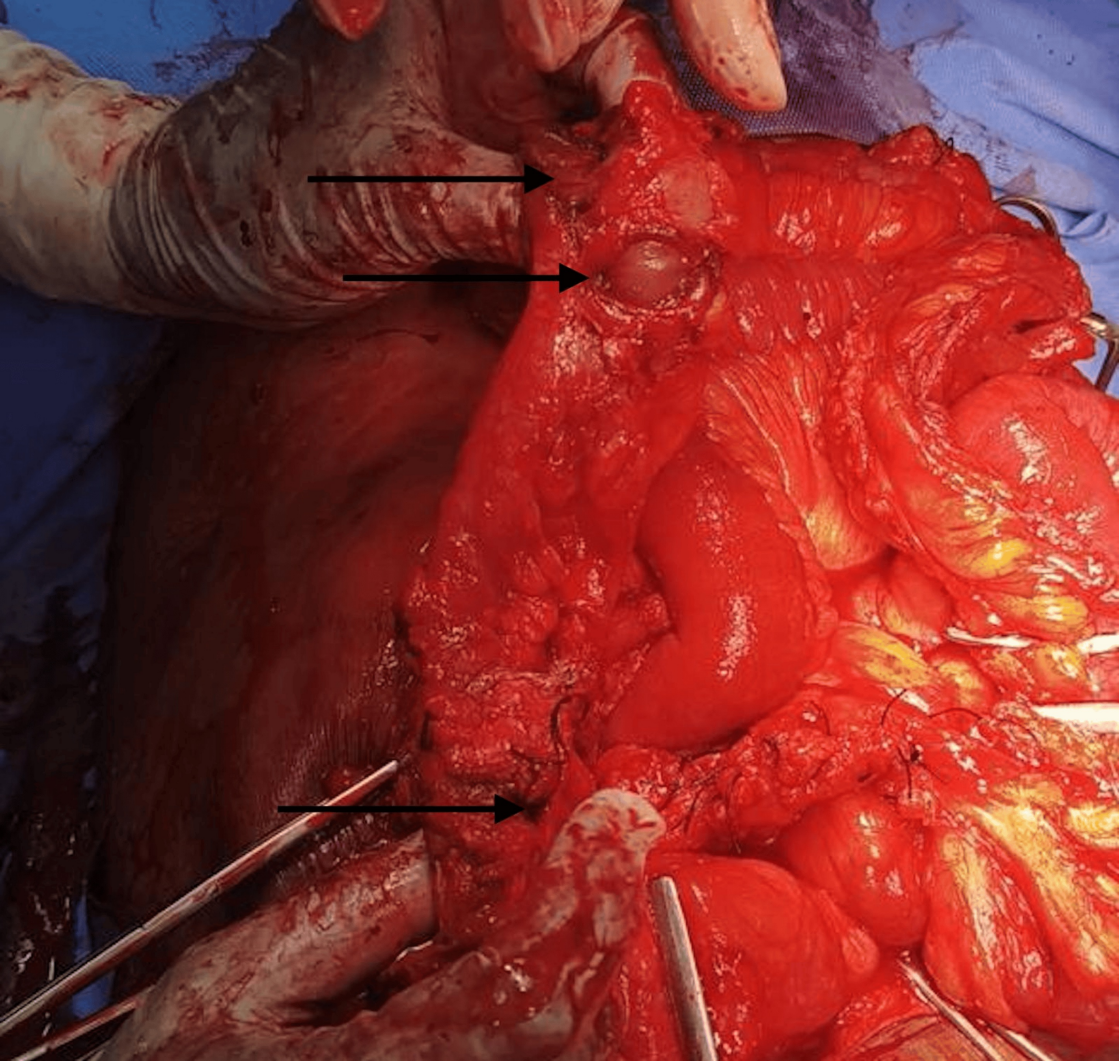
Representative image showing multiple perforations in the large intestine(pointer)

In the postoperative period, as shown in Table 4, the highest incidence of complications was wound infection, affecting approximately one-third of patients (n = 29, 29%), of which the most common causative organism was *Staphylococcus aureus*, followed by respiratory complications, including pneumonia and acute respiratory distress syndrome (ARDS), together affecting 17 patients (n = 17, 17%). Table 5 shows the distribution of mortality rates based on the affected organ, with large intestine pathology (Figure 4) having the highest proportion (n = 2, 25%). This was followed by the duodenum, with a total of five deaths out of 31 cases (n = 5, 16%). The results of the study are tabulated as follows:

**Table 4:** Relative incidence of various postoperative complications in hollow viscus perforation patients

Organ	Burst abdomen (N = 3)	Wound infection (N = 29)	Residual abscess (N = 4)	EC fistula (N = 12)	Pneumonia/ARDS (N = 17)	Sepsis (N = 10)
Gallbladder	-	-	-	-	4	1
Stomach	1	7	-	-	4	4
Duodenum	-	14	-	4	8	5
Appendix	1	-	3	6	2	2
Small intestine	-	6	-	2	1	1
Large intestine	1	2	1	-	2	2

**Table 5:** Organ-specific mortality in hollow viscus perforation CFR = (Number of deaths due to a specific disease / number of cases affected by that disease) × 100

Organ	Total no. of cases (N = 100)	No. of deaths (N = 13)	Case fatality rate (CFR)
Gallbladder	12	-	-
Stomach	20	3	15%
Duodenum	31	5	16.1%
Appendix	16	2	12.5%
Small intestine	13	1	7.6%
Large intestine	8	2	25%

## Discussion

The clinical presentation of hollow viscus perforation is often dramatic, with sudden onset of severe abdominal pain being the hallmark symptom. The pain is typically sharp, severe, and localized initially, often progressing to become diffuse as peritonitis develops. With peptic ulcer perforation, patients may describe the pain as a “knife-like” sensation. Additional symptoms and signs include fever, tachycardia, nausea, vomiting, and abdominal rigidity, followed by signs of shock. Initial laboratory tests and an abdominal X-ray are performed, followed by a CT scan to confirm the diagnosis.

In some cases, preoperative predictors, such as association with comorbidities and chronic inflammatory conditions, can help assess the severity of the outcome. A parallel study showed that hypertension and type 2 diabetes mellitus are the most common comorbidities associated with peritonitis [13]. Our study found that morbidity and mortality are highest in the older age group, which is in concordance with similar studies by Parwez et al. [14]. A study by Paryani et al. demonstrated that certain age groups, which are less than 20 years and over 50 years of age, handle stress poorly, leading to an increased risk of mortality [15].

A similar study from 2017 revealed that the incidence of hollow viscus perforation was more common in men. Duodenal ulcers were found to be the most frequent cause of perforations, which was consistent with the results in our study population [16]. Our findings were not the same as those of a 2023 study that indicated appendicular perforation as the most common etiology. Consistent with our findings, they reported that the most common presentations were abdominal pain, vomiting, and distension [17].

In select cases of small, contained perforations, particularly in patients who are poor candidates for surgery, conservative management with antibiotics and bowel rest may be attempted. However, this approach carries the risk of worsening peritonitis. Hollow viscus perforation has a high risk of complications, many of which are life-threatening if not promptly treated. The leakage of GI contents into the peritoneal cavity leads to peritonitis, which can quickly progress to sepsis and septic shock if left untreated.

In some cases, localized abscesses may form if the perforation is contained. These abscesses require drainage, either surgically or percutaneously. Chronic inflammation or delayed healing may result in the development of enterocutaneous fistulas, particularly in patients with Crohn’s disease. Bacterial contamination of the peritoneal cavity can lead to systemic infection, potentially causing multi-organ failure if not managed aggressively with antibiotics and appropriate source control.

The first limitation of this study is that it is a retrospective study, making it less accurate than a prospective study. Second, the small sample size does not provide a complete picture of the complications; therefore, a multicenter study is needed for a detailed understanding of the disease.

## Conclusions

Hollow viscus perforation is a life-threatening condition that requires prompt diagnosis and management; otherwise, it can lead to peritonitis and sepsis. This study found that duodenal and gastric ulcers are the most common causes of perforation. Hollow viscus perforation was more common in males than in females. The incidence of perforation was highest in the 41-50 age group, but mortality was greater in patients over 50 years. Abdominal pain, nausea, and vomiting were the most common presentations seen in our study population. Although risk factors such as NSAID use, *H. pylori *infection, and advanced age increase susceptibility, proper risk stratification and a well-planned treatment approach can substantially help prevent progression to perforation.
